# The Matron's Department

**Published:** 1905-11-18

**Authors:** 


### THE MATRON'S DEPARTMENT.

### XIII. FUEL AND LIGHT.

It is not probable that the matron will be called on to decide the vexed questions of how best to warm and light the institution. Nor is it possible within the narrow limits of this article to enter upon these important matters. But, except in very large establishments, it will fall to the matron's share to control the use of the appointed means and get the best possible results out of the system in force, however faulty she may consider it.

With regard to fuel, vigilance is required from the very beginning to ensure that the quantity of coal delivered corresponds with the invoice, and this is a point often neglected. Sacks must be counted and one occasionally weighed; with truck loads the weight must be tested and careful watch must be exercised to provide that the coal is of the quality contracted for and that it is not delivered wet or with an undue proportion of coal dust. These precautions may tell for many pounds in the year in large transactions. A good padlock should be affixed to the coal-cellar and the supplies must be given out once a day under the superintendence of the head porter or some trustworthy official, and the key then restored to the housekeeper. The hard coal for the engine-room should be kept in a separate cellar and the engineer's coal bunk should be replenished daily instead of his being allowed to use from the main supply.

As has been many times reiterated the only way to control is to know. It is impossible to check the consumption of coal in either a large or a small household without knowing how much should be burnt in each grate and in the engine-room for a given number of hours. The housekeeper must then set herself to find out what weight of coal will suffice to keep in each part of the establishment a good fire going as long as it is needed, and having made out a list of requirements, based on a liberal rather than a stingy scale, should let the porter have a copy, with such daily modifications as may occur, and see that the correct amount is consigned to the right place every morning. It will not, of course, be found necessary to weigh it over. The capacity of the coal-boxes in proportion to weight being once ascertained the weight of coal issued every morning, less the surplus from the previous day, will be entered by the housekeeper in a book kept for that purpose, and she will thus know from week to week what amount of coal is consumed on the premises. In mild weather less should be used. In very severe weather the supplies may be increased. The stoking of fires should not be left to chance. There should not be a roaring furnace which no one can approach alternating with a black and smouldering mass. The fires should be made up at regular intervals through the day and this duty should be entered as it recurs on the time-table of the person responsible, in each part of the house and in each ward. If the fire is never let low an equable heat will be diffused throughout the room with far less expenditure of fuel than is required by the spasmodic process of piling half a hundred-weight of coal on at a time. The housekeeper must not think it beneath her notice to bring even the fires under discipline. If she has to deal with an inferior quality of coal a little well-broken coke will help to build up bright fires, and the removal of ashes twice a day must be sedulously attended to. The good housekeeper has better resources than grumbling when compelled to use bad instruments; for they do but move her to the exercise of greater ingenuity in order to attain her end.

The wood for kindling should be issued daily or once a week, according as accommodation serves, and the housekeeper should know how many sticks are needed to light a fire with the coal in use.

On the intricacies of hot air and hot water heating we cannot here enter. Gas fires are not an economy if the cost of the gas is balanced against the cost of the coal consumed. But they are an economy of labour, and they are exceedingly useful in waiting-rooms or places where a fire is only needed for an hour or two occasionally. They need more attention than is generally supposed if they are to be satisfactory. The burners must be constantly inspected and kept scrupulously clean, and the asbestos must be relaid and renewed from time to time. Gas fires, too, are very subject to small leakages which are not only injurious to health but also a source of considerable waste. On the whole, where they are in use it is wiser to let a gas-fitter overhaul them twice a year. The same remarks apply to gas-cooking stoves, which, unless kept in perfect order are expensive and unsatisfactory. The cost of regular inspection is repaid a hundred fold.

There is no form of lighting which does not demand incessant watchfulness on the part of the housekeeper. Gas is still the commonest form of lighting in institutions although it vitiates the air and defiles the ceilings and is thus very troublesome for indoor use. Much may be done by the aid of incandescent burners to minimise these disadvantages, and with a housekeeper who will herself superintend the renewal of the mantles this form of lighting should prove perfectly satisfactory. The faint smell of gas which is often perceptible in places where by-passes are in use should be regarded as a symptom of something wrong and should be at once attended to. Scrupulous cleanliness is essential. Globes and chimneys must be washed every week and any spot above the burner which catches smoke must receive frequent attention. Incandescent light is not very good for reading or needlework unless shaded and placed at not too great a distance from the eyes. When community sitting-rooms are lighted as so often happens in the centre from the ceiling a great strain is put on the eyes, but the matron may do much to relieve the glare with softly-tinted globes and should supplement the light with two or three ordinary paraffin reading lamps. An air of comfort would thus be diffused at the smallest possible expenditure and with little trouble. The windows should at no time be altogether closed in rooms lighted by gas. Separate meters should be fixed to the nurses' quarters, medical officers' quarters, kitchen premises, and hospital building. The housekeeper must take a daily reading of the meters, and can thus tell from day to day should the gas be improperly used for warming bedrooms or left on when not required. There should be regular hours for turning it off and on at the meter, and the housekeeper, in her morning inspection of all the rooms, must note whether each tap is properly turned off, or dangerous escapes may take place. Much discomfort is avoided if the gas-jets on staircases or passages used at night are connected to a separate meter.

The use of electric light relieves many anxieties, but it does not dispense with the necessity for care in little things. The meter readings must be entered daily, as in the case of gas, and the globes must be washed once a week. The renewal of lamps, which should be done when they burn red and not be delayed till they become extinct, becomes one of the housekeeper's regular cares. Naked lamps are best for use on staircases and in passages, but for rooms ground glass is much to be preferred. The same mistakes which have been commented on with regard to ceiling lights in gas are frequently, and with less excuse, committed in sitting-rooms fitted with electric light. In this case, however, it is very easy to connect reading-lamps by cords from the centre, and the advantages to eyesight and comfort are so striking that the matron will not find it difficult to get this trifling alteration carried out. It frequently happens when electric light is newly installed that mistakes are made in the wiring: two lights, for instance, placed where, by studying turnings, one might have sufficed, some dark stair omitted, or the light awkwardly situated with regard to work necessary to be carried on in the room. It is far better economy when deficiencies of these kinds are observed to pay for alterations rather than wear out the sight of the staff or expose them to perpetual inconvenience. A little expenditure on auto matic attachments to the lavatories is soon repaid.

In some parts of the country, but in few large hospitals, the use of paraffin lamps still survives, and, unwelcome as this form of lighting may be to the matron, she may take heart from knowing that it is the least expensive form of lighting, and that its efficiency depends entirely on herself. The old-fashioned notion that servants cannot be trusted to keep lamps in good order must be dismissed altogether. It is housemaids' work, and any young woman of ordinary intelligence can be taught to do it as well as it can possibly be done. To this end the matron must arrange a place where the work can be done undisturbed, without hurry, and with proper appliances. It must be the duty of one person, who must be thoroughly instructed and encouraged to take special pride in this branch of her work. There must be constant inspection, rigid cleanliness, wholesome praise when all is well, and help in overcoming difficulties when things go wrong. Good lamps and the best oil must be procured, and burners must be renewed from time to time. With these precautions the lamps will neither smoke nor be offensive to the smell, and if a sufficient number are used the absence of gas and electricity need cause discomfort to no one. Too commonly, however, oil lamps are fixed with the greatest parsimony, and passages are often left in complete darkness which would as a matter of course be provided with a gas jet or electric burner if the system of lighting were changed.

Light and warmth are two large factors in the well-being of an institution, and the methods employed to produce them are large factors in hospital expenditure. Let it not be thought that the details enumerated above are too trifling to engage the matron's attention. In the great hospitals, where the minutest leakage in expenditure is reflected with startling effect in the bills, precautions against waste in light and fuel are adopted as a matter of course. It is in smaller establishments that a lax system is often in vogue. The matron, on whom the responsibility of these matters comes to rest, must not be contented merely with economy. She must aim at getting the maximum quantity of light and warmth for her household which the means at her disposal will permit, and unremitting attention to detail is the only road to success.

